# Relocalization of Translation Termination and Ribosome Recycling Factors to Stress Granules Coincides with Elevated Stop-Codon Readthrough and Reinitiation Rates upon Oxidative Stress

**DOI:** 10.3390/cells12020259

**Published:** 2023-01-08

**Authors:** Desislava S. Makeeva, Claire L. Riggs, Anton V. Burakov, Pavel A. Ivanov, Artem S. Kushchenko, Dmitri A. Bykov, Vladimir I. Popenko, Vladimir S. Prassolov, Pavel V. Ivanov, Sergey E. Dmitriev

**Affiliations:** 1 Belozersky Institute of Physico-Chemical Biology, Lomonosov Moscow State University, 119234 Moscow, Russia; 2 Department of Medicine, Brigham and Women’s Hospital, Harvard Medical School, Boston, MA 02115, USA; 3 Faculty of Bioengineering and Bioinformatics, Lomonosov Moscow State University, 119234 Moscow, Russia; 4 Faculty of Biology, Lomonosov Moscow State University, 119234 Moscow, Russia; 5 Engelhardt Institute of Molecular Biology, Russian Academy of Sciences, 119991 Moscow, Russia

**Keywords:** stress granules, translation termination, ribosome recycling, translation reinitiation, ETF1, GSPT1, MCTS1, ligatin, oxidative stress, upstream open reading frames uORFs

## Abstract

Upon oxidative stress, mammalian cells rapidly reprogram their translation. This is accompanied by the formation of stress granules (SGs), cytoplasmic ribonucleoprotein condensates containing untranslated mRNA molecules, RNA-binding proteins, 40S ribosomal subunits, and a set of translation initiation factors. Here we show that arsenite-induced stress causes a dramatic increase in the stop-codon readthrough rate and significantly elevates translation reinitiation levels on uORF-containing and bicistronic mRNAs. We also report the recruitment of translation termination factors eRF1 and eRF3, as well as ribosome recycling and translation reinitiation factors ABCE1, eIF2D, MCT-1, and DENR to SGs upon arsenite treatment. Localization of these factors to SGs may contribute to a rapid resumption of mRNA translation after stress relief and SG disassembly. It may also suggest the presence of post-termination, recycling, or reinitiation complexes in SGs. This new layer of translational control under stress conditions, relying on the altered spatial distribution of translation factors between cellular compartments, is discussed.

## 1. Introduction

Cell survival under stress requires the cell to mount a precise and rapid response to environmental changes [1,2,3]. As protein synthesis is a major energy-consuming process [4], its regulation under these conditions is of critical importance. While stress results in global translation repression, the synthesis of certain proteins (i.e., a stress-specific proteome) is increased [1,3]. The best-characterized feature of stress-resistant mRNAs is the presence of specific upstream open reading frames (uORFs) in their 5′ untranslated regions (5′ UTRs), allowing them to escape the shut-down of global protein synthesis [5]. However, even in the pool of uORF-containing mRNAs, only a limited portion is indeed resistant to stress [6].

The response of cells to changing environments under stress also includes the formation of specialized ribonucleoprotein (RNP) biocondensates or microscopically visible RNA granules [7]. Two major types of such cytoplasmic RNP foci have been identified: stress granules (SGs) and processing bodies (PBs) [8,9,10]. The formation of these RNPs is highly conserved among eukaryotes from yeast to multicellular animals and plants. While PBs are proposed to be associated with mRNA silencing and decay (though not required for these functions), and often present under unstressed conditions, SGs are formed specifically in response to various kinds of stresses and are directly associated with translation shutdown. Specifically, under stress-induced phosphorylation of the α subunit of translation initiation factor eIF2 [11], leading to a massive disassembly of polysomes, untranslated mRNAs are recruited to SGs (reviewed in [8,9,10,12,13,14]). Though the precise functions of SGs are still obscure, there is evidence for their contribution to cell survival under stress, as well as to antiviral defense [7,8,9,10,15].

Interestingly, while SGs and PBs are physical entities, which are microscopically visible under stress, their protein and RNA components shuttle in and out of them, and SGs themselves are highly dynamic—rapidly assembling and disassembling with the onset and release of stress (reviewed in [9]). It is thought that the fate of the mRNAs recruited into SGs is determined during the stress response, either by returning to the translationally active pool of mRNAs or by further relocalization into PBs, possibly for their storage or perhaps degradation, both of which are still hypothetical [8,16,17].

The number of proteins identified as SG constituents has been greatly expanded in the past few years. Many of them are related to translational control, mRNA turnover, cell signaling, and other cellular processes. Some of these proteins are directly involved in the regulation of SG formation, some are recruited to SGs, and some interact with SG components [18,19,20,21,22,23,24,25,26,27,28]. However, the dynamic and transient nature of SGs makes it difficult to analyze the SG content comprehensively and unambiguously, and depending on the approach, there are variations in the SG proteome outcomes.

It is well established that SGs contain untranslated mRNAs, 40S ribosomal subunits, and a number of translation initiation factors, which together could form 48S preinitiation complexes (PICs) under normal conditions. Among the factors, the multi-subunit protein eIF3, the cap-binding factor eIF4E, RNA helicase eIF4A, and scaffold protein eIF4G were reported as SG components [23,29,30] (Table 1). However, there is still no solid evidence that these factors are actually bound to the 40S subunit and mRNAs within SGs like in canonical 43/48S PICs, thus making it possible that they are part of other multiprotein complexes. Recent data, including those from high-throughput studies [23,24,25,27], indicate that translation factors found in SGs are not limited to those involved in the initiation stage. For instance, an elongation factor eIF5A was reported to be present in mammalian SGs [31,32], while termination factors Sup35p and Sup45p were detected in heat-shock-induced SGs in yeast [33]. Since all phases of translation are intrinsically inter-connected functionally and even spatiotemporally, it is possible that other translation factors, beyond the well-characterized proteins involved in translation initiation, also contribute to SG biology. Here, we directly probed whether translation termination and ribosome recycling factors in higher eukaryotes are stress-responsive and SG biology-related.

Translation termination in eukaryotes is mediated by two release factors, eRF1/Sup45p and eRF3/Sup35p [34,35]. Once a termination codon enters the ribosomal A-site, eRF1 is delivered to the complex by the GTPase eRF3. This is followed by peptidyl-tRNA hydrolysis and nascent peptide release, thus forming the 80S post-termination complex [36,37]. The activity of eRF1 is stimulated by the ABCE1/Rli1p protein [38,39], which also plays a crucial role in the downstream events. Importantly, the depletion of eRF1 and eRF3 in mammalian cells leads to inefficient translation termination and increased levels of stop-codon readthrough [40,41].

**Table 1:** Localization of translation machinery components in SGs.

	Cellular Component	Localization in Mammalian SGs	References	Remarks
	poly(A)+ mRNA	Yes	[29]	
	40S	Yes	[29] and many more	
	60S	No	[29,42,43]	
	PABP	Yes	[44] and many more	
Translation initiation	eIF1	No data		In [45] eIF1 was mentioned as detected in SGs (as an unpublished observation of the authors).
	eIF1A	No data		Yeast eIF1A localizes to SGs under certain, but not all, stress conditions [33,46,47].
	eIF2	Diverse or controversial	[29,43,48]	“Canonical” SGs lack eIF2, although under some conditions eIF2 was found in SGs (for review, see [8]).
	eIF2B	Diverse or controversial	[29,43]	Yeast eIF2B was reported to reside in large cytoplasmic foci even in the absence of stress [49].
	eIF3	Diverse	[29] and many more	eIF3 is absent in UVC- or selenite-induced SGs [32,50] but present in the “canonical” SGs (for review, see [8]).
	eIF4A	Yes	[23,25,30,32,48]	Two paralogs (eIF4A1 and eIF4A2), encoded by different genes are both localized to SGs.
	eIF4E	Yes	[29] and many more	
	eIF4G	Yes	[23,29,32]	Two paralogs (eIF4GI and eIF4GII), encoded by different genes, are both localized to SGs.
	eIF4B	Yes	[48,51]	
	eIF4H	Yes	[25]	
	eIF5	No	[29]	
	eIF5B	No data		Yeast eIF5B localizes to NaN_3_-induced SGs [47].
	eIF2A	Controversial	[23,52]	According to mass-spectrometry data, eIF2A associates with SG; however, immunofluorescence data contradicts this.
Elongation	eEF1A	No data		Yeast eEF1A is excluded from the heat-shock induced SGs [33].
	eEF1B	No data		Yeast eEF1Bγ is present in the heat-shock induced SGs [33].
	eEF2	No	[43]	Yeast ortholog of eEF2 (Eft1p) was reported to absent from SGs [33].
	eIF5A	Yes	[31,32]	
Termination	eRF1	This study		Yeast eRF1 (Sup45p) was reported to localize to heat-shock induced SGs [33].
	eRF3	This study		Yeast eRF3 (Sup35p) was reported to localize to heat-shock induced SGs [33].
Recycling	ABCE1	This study		No information about localization of the yeast orthologs (Rli1p, Tma64p, Tma20p, and Tma22p, respectively) is available as well
	eIF2D	Yes & This study	[52]	
	MCT-1	This study		
	DENR	This study		

The subsequent step of the translation cycle is ribosome recycling, initiated by ABCE1/Rli1p-catalyzed dissociation of the 80S ribosome into mRNA-bound 40S and free 60S subunits [38,53,54]. In cooperation with eRF1, ABCE1 promotes both the termination and displacement of the large subunit, thus bridging the two final stages of the translation cycle [36,37,55]. The resulting 40S•tRNA•mRNA complex is disassembled by eIF2D/Tma64p or a heterodimer of MCT-1/Tma20p and DENR/Tma22p proteins [56,57]. Upon recycling, individual components of the post-termination complex are released and available for new translation rounds (reviewed in [36,37,58]).

In in vitro systems, eIF2D can release tRNA and mRNA from the post-termination complex [56,59], while in some special cases, it can also stabilize the 40S•tRNA•mRNA complex during non-canonical translation initiation [56,60] or promote reinitiation [59]. The C-terminal part of eIF2D is represented by the SUI1 domain, which is also found in translation factors eIF1 and DENR. eIF1/Sui1p is a translation initiation factor that binds to the P-site of the 40S subunit and plays a major role in ribosomal scanning and start codon selection [61]. DENR, which forms a heterodimer with MCT-1 both in mammalian and yeast cells [62,63], binds to the ribosomal P-site similarly to eIF1 [64,65]. The functions of the MCT-1•DENR heterodimer are apparently redundant with those of eIF2D, at least in part, since double knockouts of their orthologs, *TMA20* and *TMA64* or *TMA22* and *TMA64*, produce a synthetic slow-growth phenotype in yeast [66]. In the mammalian in vitro translation reconstitution system, their activity in initiation and recycling is almost identical [56]. The absence of Tma64p (eIF2D) and Tma20p•Tma22p (MCT-1•DENR) in yeast cells leads to the inefficient release of the post-termination 40S ribosome from mRNA and promotes the translation reinitiation at uORF-containing mRNAs [57]. The importance of eIF2D, MCT-1, and DENR for translation reinitiation at uORF-containing mRNAs is also well established in fly and mammalian cells [67,68]. Thus, the major function of these proteins could be control of the P-site tRNA in recycling and reinitiating 43S complexes, although their precise roles in vivo remains uncertain. Their possible contribution to SG biology has also not been investigated to date (Table 1).

Upon severe stress (e.g., sodium arsenite-induced oxidative stress), the translatome of mammalian cells changes dramatically (see [1,2,6] and references therein). However, whether translation termination and ribosome recycling efficiencies are stress-responsive and whether the subcellular localization of corresponding translation factors under stress is affected remains unknown. Here, we directly investigated a possible stress-induced modulation of the termination/recycling stage of translation. Surprisingly, we detected elevated rates of translation reinitiation and stop-codon readthrough under stress conditions triggered by arsenite, and observed partial recruitment of translation termination factors (eRF1 and eRF3) and recycling factors (ABCE1, MCT-1, DENR, and eIF2D) into SGs, but not PBs. Our data suggest that stress strongly affects translation termination, reinitiation, and ribosome recycling in live cells. We also show that the localization of translation/recycling factors may partially contribute to translation termination and recycling events.

## 2. Materials and Methods

### 2.1. Cell Culture and Arsenite Treatment

HEK293T, U2OS, and HeLa cell lines (ATCC, Manassas, VA, USA) were grown in DMEM (Gibco, New York, NY, USA) supplemented with 10% FBS (HyClone, Logan, UT, USA) in the presence of penicillin and streptomycin (Paneco, Russia) in a humidified 5% CO_2_ atmosphere at 37 °C. For immunostaining, the cells were cultured to ~70% confluence on coverslips. For ICC assays, HeLa or U2OS cells were exposed to 250 µM sodium arsenite (Sigma-Aldrich, St. Louis, MO, USA) for 1 h and then immediately fixed. For puromycin/emetine assays, U2OS cells were treated with 250 µM sodium arsenite for 90 min with the addition of either puromycin (10 µg/mL) (Sigma-Aldrich) to promote SGs or emetine (10 µg/mL) (Sigma-Aldrich) to disassemble SGs after 30 min of arsenite treatment.

### 2.2. Antibodies and Immunocytochemistry

The following primary antibodies were used: rabbit polyclonal anti-eIF2D, anti-eRF1, and anti-eRF3 (Proteintech Group, Chicago, IL, USA: 12840-1-AP, 10884-1-AP, and 12989-1-AP, respectively), anti-ABCE1 (MyBioSource, California, CA, USA, MBS9128630), anti-MCT-1 (Abcam, Waltham, MA, USA, ab135593). Mouse anti-G3BP1 (H-10) (Santa Cruz Biotechnology, Dallas, TX, USA, sc-365338), and goat anti-eIF3b (N-20) (Santa Cruz Biotechnology, sc-16377) were used to detect SGs, and mouse anti-p70 S6 kinase, which exhibits cross reactivity with Hedls [69], was used to detect PBs.

Rabbit IgG-specific secondary antibodies conjugated to Alexa Fluor 555 or mouse IgG-specific secondary antibodies conjugated to Alexa Fluor 488 for ICC in HeLa were obtained from Invitrogen, USA (A32732, A32723); anti-goat-Cy5, anti-mouse-Cy2, and anti-rabbit-Cy3 secondary antibodies from Jackson ImmunoResearch Laboratories, USA, were used for ICC in U2OS cells. 

Cells were grown on glass coverslips for 12 to 24 h, fixed with absolute methanol for 5 min at −20 °C, and post-fixed with 3% paraformaldehyde (Sigma-Aldrich) for 15 min at +4 °C. Cells were permeabilized with 0.5% Triton X-100 for 5 min at RT prior to blocking (10% FBS with 0.1% glycine (Sigma-Aldrich) in PBS) for 1 h at RT. Cells were then incubated in primary antibodies for 1 h at RT or overnight at +4 °C, followed by washing with PBS (3 × 5 min), secondary antibody incubation (1 h at RT), and washing with PBS (3 × 5 min). After immunostaining, the coverslips were mounted using polyvinyl mounting media or Aqua-Poly/Mount with DAPI (Polysciences Inc., Warrington, PA, USA).

### 2.3. Microscopy

Microscopy for HeLa cells was performed using an Olympus IX51 microscope supplied with a 14-bit Olympus XM10 camera and UniBlitz D122 shutter driver (Olympus, Tokyo, Japan) controlled by Micromanager software (v.1.4.22), or with the Axiovert 2000 M microscope (Carl Zeiss, Jena, Germany) using 63× and 100× Plan-NEOFLUAR objectives. Images were acquired with AxioVision 3.1 software (Carl Zeiss) using the AxioCam HRc digital video camera (Carl Zeiss). Microscopy for U2OS ICC was performed using a Nikon Eclipse E800 microscope (Nikon, Japan) with a 63× Plan Apo objective lens (NA 1.4) and illuminated with a mercury lamp and standard filters for Cy2 (FITC HQ 480/40; 535/ 50), Cy3 (Cy 3HQ 545/30; 610/75), and Cy5 (Cy 5 HQ 620/60; 700/75). Images were taken with a Pursuit Monochrome 1.4 MP Digital Camera (Diagnostic Instruments, Heights, MI, USA) using the manufacturer’s software (SPOT Software, Version 5.2). Raw TIF files were processed with ImageJ 1.52p (NIH, USA) or Adobe Photoshop CS5 software (Release 21.2.0, Adobe Systems, Mountain View, CA, USA). 

### 2.4. In Vitro Transcription

The m^7^G-capped and polyadenylated mRNAs encoding firefly (Fluc), *Renilla* (Rluc), and NanoLuc (Nluc) luciferases were prepared as previously described [57,70,71]. For convenience, *no_uORFluc*, *uORF1luc*, *RFluc2*, and *RF1luc1fus* constructs from [57] hereafter are named *Fluc*, *REI uORF-Fluc*, *REI Rluc-Fluc*, and *control Rluc-Fluc*, respectively. Plasmids encoding NanoLuc variants, having the Nluc coding region (CDS) extended at the 5′ end by 39 nt derived from the human *HBB* gene [71], were kindly provided by Elena Alkalaeva (IMB RAS) and Ilya Terenin (MSU). The CDS extension was fused in-frame to the Nluc CDS either directly or through termination codons UAG or UGA. Partial mRNA sequences are shown in Supplementary Table S1. In vitro transcription was performed with the RiboMAX kit (Promega, Fitchburg, WI, USA). The resulting transcripts were precipitated with 2M LiCl and capped with the Vaccinia Capping System (New England Biolabs, Ipswich, MA, USA), followed by another LiCl precipitation. All mRNA transcripts were checked for integrity by denaturing urea polyacrylamide gel electrophoresis. 

### 2.5. Fleeting mRNA Transfection (FLERT)

Short-term mRNA transfection of HEK293T, HeLa, or U2OS cells was performed as described previously [72]. Briefly, cells were cultured in 24-well plates to ~70% confluence. Sodium arsenite was added to the medium to the indicated concentration 1 h before mRNA transfection. The cells were transfected with reporter mRNAs using GenJect-U (Molecta, Moscow, Russia). Two hours after transfection, cells were harvested, and luciferase activities were analyzed using the Dual Luciferase Assay kit (Promega). All transfections were repeated at least three times in different cell passages. The mean values ±SD were calculated.

## 3. Results

### 3.1. Translation Reinitiation Rate Is Increased under Conditions of Arsenite-Induced Stress

While oxidative stress-induced changes in translation initiation and elongation are well studied [1,2], the effects on the other two stages of the translation cycle, translation termination and ribosome recycling, have never been systematically analyzed. Dysregulated termination efficiency can be recognized by assessing the fidelity of stop codon recognition, while perturbations in ribosome recycling can be detected by the altered translation reinitiation rate. Thus, we used three previously described mRNA reporter systems [57,71] to assess the stop-codon readthrough and translation reinitiation rates in human HEK293T cells treated with different concentrations of ortho-arsenite (NaH_2_AsO_3_, sodium dihydroarsenite), a commonly used inducer of oxidative stress [6,29,72,73,74,75]. Fleeting mRNA transfection (FLERT) [72] was used for the rapid delivery of the reporters into cultured cells 1 h after stress induction.

**Figure 1:**
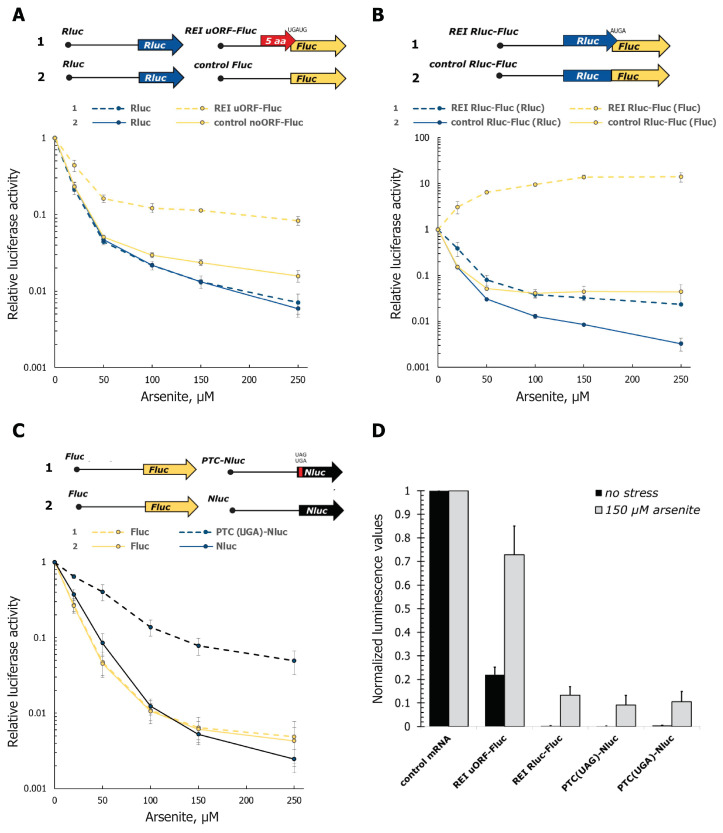
The rates of translation reinitiation and stop-codon readthrough are increased under conditions of arsenite-induced stress, as revealed by luciferase reporter assays. (**A**–**C**) Schematic representation of reporter mRNA constructs (upper panels) and relative luciferase activities in arsenite-treated HEK293T cells transfected with the constructs, normalized to that in untreated cells (lower panels). Reporter mRNAs have either a short uORF (**A**) or the full-length Rluc-encoding ORF (**B**) in front of *Fluc* CDS or harbor a PTC (premature termination codon) in the main (*Nluc*) CDS (**C**), as described in the text. In (**A**) and (**C**), transcripts encoding Rluc or Fluc, as indicated, were co-transfected as controls; 2 h after transfection, cells were lysed, and reporter-dependent luciferase activities were measured. (**D**) Translation efficiencies of the reporter mRNAs, divided into those of the corresponding control transcripts after normalization, in untreated or 150 µM arsenite-treated cells are shown (calculated from the same data as in (a–c)). The mean values (±SD) of at least three independent experiments are shown. The absolute values of luciferase units for untreated cells were ~9 × 10^5^ for *control Fluc*, ~2 ×10^5^ for *REI uORF-Fluc*, ~3 × 10^6^ for *Rluc*, ~4 × 10^5^ for *control Rluc-Fluc* (Fluc), ~3 × 10^3^ for *REI Rluc-Fluc* (Fluc), ~1 × 10^7^ for *Nluc*, ~4 × 10^4^ for both *PTC-Nluc* mRNAs, ~8 × 10^5^ for *Fluc*; the background (no mRNA) values were 50 units for Fluc and 120 units for Rluc and Nluc.

The first system (Figure 1A, Supplementary Table S1) was used to measure reinitiation rates [57]. It consisted of capped and polyadenylated in vitro synthesized mRNAs encoding firefly luciferase (Fluc) with either a 37-nt artificial 5′ UTR (*control Fluc*) or the same 5′ UTR with a 5-codon long uORF joined out-of-frame with the *Fluc* CDS (coding region) by the AUGA start-stop sequence (*REI uORF-Fluc*). *Renilla* luciferase mRNA (*Rluc*) with a simple 5′ UTR leader was co-transfected with the *Fluc* reporters for normalization. Two hours after transfection, cells were harvested and luciferase activities were measured. In our experiments, we used a range of arsenite concentrations to induce moderate to severe oxidative stress [74,76], namely 20, 50, 100, 150, or 250 µM (Figure 1A–C). 

As expected, the presence of the uORF in the 5′ UTR reduced the translation efficiency of the *REI uORF-Fluc* mRNA, as compared to the *control Fluc* reporter (Figure 1D, compare black bars). Arsenite treatment induced a concentration-dependent decrease in the translation activity of all mRNA reporters (Figure 1A), resulting in the production of ~65-fold less luciferase activity by the *control Fluc* mRNA reporter in cells treated with 250 µM NaH_2_AsO_3_. However, the inhibitory effect of arsenite was substantially diminished in the case of the uORF-containing mRNA, where only a ~12-fold decrease was observed (Figure 1A). In an intermediately high (150 µM) concentration of arsenite, the inhibitory effect of uORF dropped down more than 3-fold, from 4.5 to 1.3 times only (Figure 1D). Similar effects were observed in HeLa (Supplementary Figure S1) and U2OS cells (see below). A possibility of Fluc production via frameshifting at the uORF or beginning of the Fluc CDS can be excluded, as in this case, its efficiency should be more than 20%, which has not been observed for random sequences previously. 

This suggests that the ribosome’s ability to initiate translation at the main *REI uORF*-*Fluc* start codon after passing over the uORF is considerably increased under conditions of stress. This could be either due to a relaxed uAUG recognition (leading to leaky scanning and/or 43S sliding through the uORF [77,78], or due to an elevated reinitiation rate (i.e., an increased ability of the post-termination ribosome to resume scanning and reach the *Fluc* start codon) [79].

To exclude a contribution of leaky scanning and 43S sliding, we used a bicistronic reporter mRNA with the full-length *Rluc* coding region instead of the short uORF, joined by the UGAUG sequence to the *Fluc* CDS (*REI Rluc-Fluc*, Figure 1B, Supplementary Table S1). *Rluc* CDS harbors >20 out-of-frame AUGs and thus effectively precludes scanning 43S complexes from reaching the *Fluc* start codon. In this case, ribosomes can reach the *Fluc* coding region only via reinitiation after termination at the *Rluc* stop codon [57]. As a control, in this experiment, we used a reporter mRNA with the same leader, but *Rluc* and *Fluc* fused into a single long ORF (*control REI Rluc-Fluc*).

Arsenite treatment resulted in a decreased production of Rluc from both mono- and bicistronic mRNA reporters (Figure 1B), although in the former case, the inhibition was somewhat more pronounced (likely due to additional arsenite effects on stability or activity of the Rluc protein in the context of Rluc-Fluc fusion). In accordance with this, Fluc production from the monocistronic mRNA was also severely repressed upon arsenite stress. Importantly, the result was drastically different for the bicistronic reporter. In this case, Fluc synthesis was not decreased but rather markedly (up to >10-fold) increased in the arsenite-treated cells (Figure 1B). The relative Fluc translation rate (from bicistronic *REI Rluc-Fluc*, as compared to monocistronic *control Rluc-Fluc* after normalization to Rluc activity), which reflects the reinitiation rate, changed even more dramatically, >40-fold, rising from ~1:325 in unstressed cells (which is close to the expected one [70]) to ~1:8 after the treatment with 150 µM arsenite (Figure 1D). Similar effects were obtained in HeLa (Supplementary Figure S1) and U2OS cells (see below).

These results clearly indicate that the proportion of ribosomes reinitiating after the first ORF translation robustly increased under conditions of arsenite-induced oxidative stress. Similar effects were observed earlier in mutant yeast strains with the deletion of *RLI1*, *TMA64*, *TMA20,* or *TMA22*, the orthologs of human genes encoding ribosome recycling factors ABCE1, eIF2D, MCT-1, and DENR, respectively [54,57].

### 3.2. Stop-Codon Readthrough Rate Is Increased under Conditions of Arsenite Stress

We further investigated whether a rate of stop-codon readthrough is affected by arsenite-induced oxidative stress. We used two reporter mRNAs (Figure 1C, Supplementary Table S1) encoding NanoLuc luciferase (Nluc) appended with a short (13-aa long) N-terminal extension derived from the human β-globin. The CDS extension was joined to Nluc CDS either directly or through a premature termination codon (PTC) [71]. Fluc-encoding mRNA with the human β-actin mRNA leader was used as a control.

As expected, PTC strongly diminished Nluc production in mRNA-transfected cells under both normal and stress conditions (Figure 1D, black bars). However, we observed a dramatic difference in the effects of arsenite-induced stress on Nluc production from the reporter mRNAs. While the translation of the *wt* Nluc mRNA produced 2.5-fold to ~400-fold less luciferase activity in cells treated with the various concentrations of arsenite we tested, the PTC(UGA)-containing mRNA showed only a 1.5-fold to ~20-fold decrease in translation under the same conditions (Figure 1C). This was equivalent to the drop of the PTC inhibitory effect from ~200-fold to only ~10-fold with a 150 µM concentration of arsenite, commonly used in stress response studies (Figure 1D). Similar results were obtained with the construct bearing a premature UAG codon (Figure 1D), as well as in other cell lines (Supplementary Figure S1, see also below). As the stop-codon readthrough rate is a proxy of the translation termination fidelity, these data indicate that arsenite-induced oxidative stress makes termination less efficient.

### 3.3. Arsenite-Induced SGs Contain Translation Termination Factors in Mammalian Cells 

During arsenite-induced oxidative stress, the overall translation inhibition caused by the phosphorylation of initiation factor eIF2α triggers SG formation and increases the number of PBs in stressed cells [8,9,44,80,81]. Previously, GFP-fused yeast translation termination factors Sup45p (eRF1) and Sup35p (eRF3) were shown to be recruited into heat-shock-induced SGs [33] and also found in PBs [82,83]. This prompted us to examine whether mammalian translation termination factors could localize to SGs as well.

**Figure 2:**
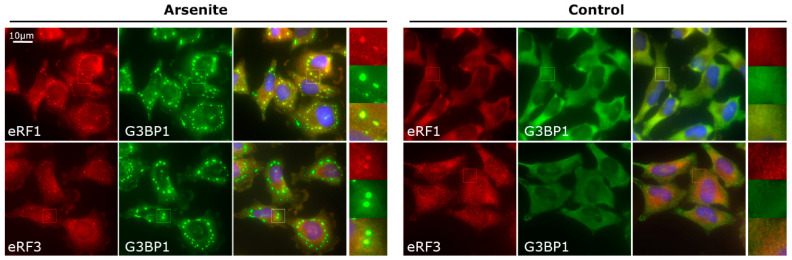
Translation termination factors are recruited to arsenite-induced SGs. HeLa cells were treated with 250 µM sodium arsenite for 1 h (left panel) or left untreated (right panel), followed by fixation and immunostaining for eRF1 or eRF3 (red false color), as indicated, and an SG marker G3BP1 (green channel). A few representative cells are shown; boxes indicate magnified areas. All large images are of the same magnification (scale bar: 10 μm).

We exposed HeLa cells to 250 µM sodium arsenite and analyzed the subcellular distribution of termination factors eRF1 and eRF3, as well as a core SG marker G3BP1, by immunofluorescence (IF) staining. In mammals, eRF1 is encoded by a single *ETF1/ERF1* gene, while eRF3 is encoded by two slightly different paralogs, *GSPT1*/*ERF3A* and *GSPT2*/*ERF3B*. In HeLa cells, we were able to detect IF signals with antibodies against eRF1 and the *GSPT2*-encoded protein (the latter could likely recognize both eRF3 variants due to their similarity). We found that eRF1 and eRF3 were diffusely distributed in the cytoplasm under normal conditions but recruited into G3BP1-positive foci under arsenite stress (Figure 2). In the latter case, staining of the remaining cytoplasm became fainter, so we concluded that eRF1 and eRF3 were partially sequestered to SGs under oxidative stress. We observed a similar relocalization of eRF1 and eRF3 in HeLa cells constitutively expressing canonical SG marker PABPC1 fused to GFP (EGFP-PABP), which is absent from PBs as reported before [8,16] (Supplementary Figure S2).

### 3.4. Ribosome Recycling and Reinitiation Factors Are Partially Recruited to SGs upon Stress

**Figure 3:**
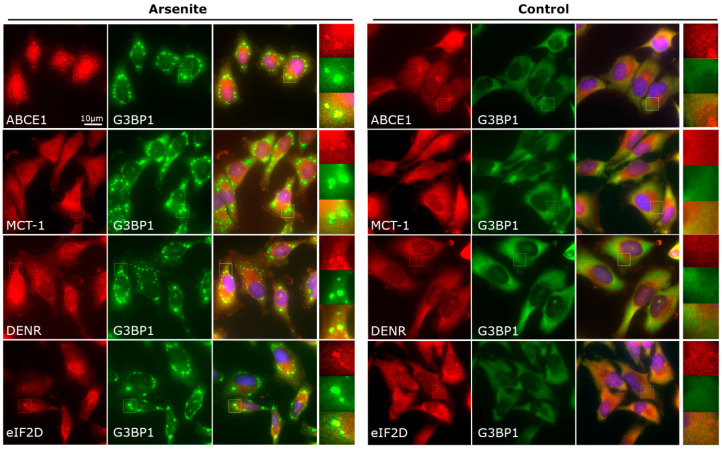
Ribosome recycling factors are recruited to arsenite-induced SGs. HeLa cells were treated with 250 µM sodium arsenite for 1 h (left panel), followed by fixation and immunostaining for the protein of interest along with an SG marker G3BP1. Immunostaining of non-treated cells is presented in the right panel. A few representative cells are shown; boxes indicate magnified areas. All large images are of the same magnification (scale bar: 10 μm).

As we found that arsenite-induced SGs contain translation termination factors, we then explored whether the ribosome recycling factors are also present in SGs. First, we examined the localization of ABCE1, an ATPase participating in peptide release, 60S dissociation, and reinitiation [38,53,54], by immunostaining HeLa cells (Figure 3). We found that, under stress, ABCE1 was markedly enriched in SGs (see below). 

We then analyzed the subcellular localization of other ribosome recycling factors, i.e., eIF2D, MCT-1, and DENR, which promote tRNA and mRNA release from the post-termination 48S complexes [56,57]. In accordance with previously reported data [30,52,60], we found that eIF2D was diffusely distributed in the cytoplasm under normal conditions, but after arsenite treatment, it congregated into SGs (Figure 3). Similarly, both MCT-1 and DENR were partially localized to SGs under stress conditions (Figure 3), although substantial portions of the proteins were still diffusely distributed in the cytoplasm. All proteins analyzed also showed similar partial localization into SGs in HeLa cells expressing EGFP-PABP (Supplementary Figure S3).

### 3.5. The G3BP1-Positive Foci Containing Termination and Recycling Factors Are Bona Fide SGs and Not PBs

To further exclude cell type or cell line specificity of the observed phenomena and explore whether the termination and recycling factors may be recruited to PBs, we performed similar IF (immunofluorescence) experiments with human osteosarcoma U2OS cells (a common model to study SGs and PBs), using SG- and PB-specific markers (Supplementary Figure S4). We confirmed the presence of eRF1, eRF3, ABCE1, eIF2D, MCT-1, and DENR in SGs, but importantly showed no co-localization with PBs detected by a genuine PB marker, Hedls/sk1. 

SGs are dynamic and translationally modulated foci that are in the equilibrium with polysomes. To further confirm that the termination/recycling factor-positive foci are *bona fide* SGs, we treated U2OS cells with arsenite in the presence of small-molecule drugs, which are known to affect SG dynamics. Puromycin and emetine, two translation inhibitors with different mechanisms of action [84], promote or partially disassemble SGs, respectively [85]. In agreement with the previous IF data, we observed prominent co-localization of both eRF1 and eIF2D with G3BP1 in puromycin-treated stressed cells, which display enlarged SGs. In contrast, emetine treatment decreased the size and abundance of eRF1/eIF2D/G3BP1-positive foci causing eRF1 and eIF2D re-distribution into the cytosol (Supplementary Figure S6). These results confirmed that mammalian translation termination and ribosome recycling factors are recruited to SGs [8].

### 3.6. Stress-Induced Modulation of Translation Termination and Reinitiation Is Enhanced in ddG3BP1/2 Cells

The depletion of eRF1 and eRF3 in mammalian cells causes effects similar to those we observed in our study [40,41], while the removal of either Rli1p or Tma64p, Tma20p, and Tma22p proteins from yeast cells was shown to up-regulate reinitiation, akin to our observations as well [54,57]. We hypothesized that the recruitment of termination and ribosome recycling factors to SGs during stress may lead to their sequestration from the cytosol and thus reduce their availability for translating ribosomes. These changes may then trigger an up-regulation of stop-codon readthrough and translation reinitiation rates, as we detected in our reporter assays (Figure 1). 

To determine whether such sequestration of translation factors into SGs contributes to the changes in translation fidelity, we assayed readthrough and reinitiation rates in U2OS ddG3BP1/2 cells, which have *G3BP1* and *G3BP2* genes knocked-out [86]. These cells respond to arsenite treatment by eIF2α phosphorylation and translation inhibition similarly to wild-type U2OS cells but do not form microscopically visible SGs [86,87].

**Figure 4:**
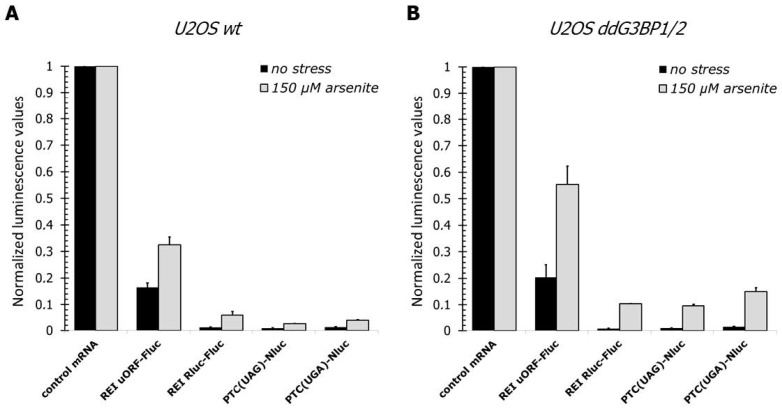
Changes of translation reinitiation and termination in arsenite-treated wt and ddG3BP1/2 U2OS cells. Wt (**A**) and ddG3BP1/2 (**B**) U2OS cells were transfected with the same reporter mRNAs as in Figure 1. Two hours after transfection, cells were lysed, and luciferase activities were measured and normalized, as described above. Translation efficiencies of the reporter mRNAs were normalized by dividing by the translation efficiencies of the corresponding control transcripts, in untreated or 150 µM arsenite-treated cells. The mean values (±SD) of at least three independent experiments are shown. The absolute values were roughly similar to those indicated in the legend in Figure 1 (and were at least two orders of magnitude above the background).

Unlike the prediction of the sequestration model, we determined an approximately 2-fold increase in the readthrough and reinitiation rates under stress conditions in the mutant cells, as compared to the wt U2OS cells (Figure 4A,B), suggesting microscopically visible SGs are not absolutely required for stress-induced readthrough and reinitiation. Thus, although a relationship between translation termination/recycling reprogramming exists, SGs only partially contribute to such a phenomenon. 

In arsenite-treated ddG3BP1/2 cells, PBs form but SGs do not [86]. Consistent with eRF1′s recruitment to SGs, we do not find evidence of its recruitment to PBs in ddG3BP1/2 cells (Supplementary Figure S5).

We cannot exclude the possibility that despite the lack of microscopically visible SGs in the ddG3BP1/2 cells, SG seeds [27] or some RNA-binding proteins could interact with the factors and/or their ribosomal complexes, and may be in association even in the absence of SGs. For example, proximity labeling studies identified submicroscopic pre-stress seeds that contain SG and PB components including some factors analyzed here [27]. It is possible that such association with SG seeds may contribute to the translation dynamics we observed.

## 4. Discussion

It is well established that SGs contain a number of translation initiation factors, 40S ribosomal subunits, untranslated mRNAs recruited from disassembled polysomes, many RNA-binding proteins, and additional components affecting mRNA metabolism [8,10,26,28,88]. It has been postulated that a stalled, translation-arrested 48S-like PIC represents a core constituent of mammalian SGs [29,43], based on the presence of translation initiation factors eIF3, eIF4A, eIF4E, eIF4G, and PABP in SGs. However, accumulating data from the multiple high-throughput studies [23,24,25,27] suggest that many other components of translation machinery could also be SG constituents, although this information is still incomplete and even controversial.

In this study, we found that proteins involved in the distinct steps of the translation cycle, i.e., the peptide chain release factors eRF1 and eRF3, as well as recycling and reinitiation factors ABCE1, eIF2D, MCT-1, and DENR, are also present in SGs formed under conditions of arsenite-induced oxidative stress in mammalian cells. A search of the RNA granule database [26] reveals that our results are consistent with data from various high-throughput studies, as most of the proteins analyzed here have been detected in SGs in at least one of such analyses. However, direct IF-based detection of their presence in SGs has not been performed before.

eRF1 and eRF3 bind to a pre-termination 80S ribosome complex at a stop codon in the A-site, in contact with primarily the 60S subunit (for review, see [36,37]). As 60S subunits are excluded from SGs [29,42,43], it is unlikely that termination factors reside in SG in complexes with ribosomes. However, they can be recruited to the compartment via interactions with other proteins or RNAs. It is known that yeast and mammalian eRF3 are known to interact with PABP, one of the core SG components [89,90,91], while eRF1 forms an evolutionarily conserved complex with eIF2 and eIF5 [92]. Moreover, both yeast Sup35p and mouse eRF3b were shown to co-localize and interact (physically and genetically) with Pub1p/TIA-1, the conserved SG component, in the yeast model [93,94]. These interactions are likely independent of the process of peptide release and do not require the ribosome or active translation, thus explaining how eRF1 and eRF3 can be recruited to SGs. Alternatively, the factors could be delivered to SGs by polysomes transiently associated with the SG “shell” [95,96] and then be confined there by protein-protein interactions. Nevertheless, localization of translation termination factors in SGs could have an important physiological role, such as facilitating a resumption of mRNA translation after stress relief and SG disassembly.

Intriguingly, we found that, under the conditions we used, the eRF1 and eRF3 relocalization to SGs coincides with a dramatic acceleration of stop-codon readthrough. In general, readthrough events are likely associated with slowed termination dynamics at the stop codon [97,98]. In this regard, it would not be surprising if termination fidelity decreased with eRF1 or eRF3 depletion [40,41]. Thus, we hypothesize here that the functional alterations we observed in arsenite-treated cells could be related to a decrease in the concentration of available eRF1 and eRF3 due to their sequestration into SGs. Alternatively, stress-induced modifications of termination factors or some other components of translation machinery may cause this effect [99]. Although our study did not show a causal relationship between the relocalization of eRF1 and eRF3 into SGs and the up-regulated readthrough (see also discussion below), this relationship is of great interest for further investigation.

The modulation of termination fidelity observed in our study may contribute to translational reprogramming of the mammalian cells under stress. It is well established that C-terminally extended isoforms of many proteins diversify their functionality by the alteration of their subcellular localization or activity [100,101]. Recent transcriptome-wide analyses based on the ribosome profiling revealed a pervasiveness of readthrough events in mammalian, fly, and yeast mRNAs, as well as changes in the stop codon recoding rate under different stress conditions (for a review, see [102]). In particular, elevated levels of stop-codon readthrough were reported in response to oxidative stress in yeast [103] and during oxygen and glucose deprivation in mammalian cells [104]. The finding we made in arsenite-treated mammalian cells is consistent with these data and suggests that it may be an important component of the cellular stress response.

One could suggest that our data obtained with the PTC-containing mRNAs may be partially explained by changing the transcript stability under conditions of stress. Indeed, altered nonsense-mediated mRNA decay (NMD) activity has been reported for arsenite-treated cells in some cases [105,106]. However, in our experiments, we did not find any indication of PTC-containing transcript stabilization, as we observed very similar effects of the arsenite stress on the translation of all our reporters at different time points after transfection (data not shown). The opposite effects of arsenite treatment on Fluc and Rluc synthesis from the same bicistronic *REI Rluc-Fluc* mRNA (Figure 1B) also contradicted this hypothesis. Thus, we concluded that either our exogenous, in vitro transcribed mRNAs were likely poor NMD substrates, or no such effects took place under the specific conditions we used.

We further showed that the ribosome recycling and reinitiation factors ABCE1, eIF2D, MCT-1, and DENR also reside in SGs in arsenite-treated mammalian cells. ABCE1 participates in three consecutive stages of the translation cycle, bringing together peptide release, ribosome recycling, and translation initiation steps [55,107,108,109]. The other three factors are thought to control post-termination 40S removal and reinitiation events, although they may also be involved in non-canonical translation initiation [56,57,60,67,68]. All these proteins are known to bind the small ribosomal subunit [64,65,108], so one cannot exclude the possibility of their presence in SGs in a form of stalled 48S post-termination or reinitiation complexes. The presence of such complexes was previously suggested for non-canonical SGs formed in under ultraviolet irradiation or selenite-treated cells [8].

DENR, MCT-1, and eIF2D have been reported to regulate the translation of uORF-containing mRNAs in yeast, mammals, and flies [57,67,68,110,111,112]. In accordance with this, we found increased rates of translation reinitiation of the reporter mRNAs with short uORF or the full-length *Rluc* coding region, preceding the reporter *Fluc* CDS, under stress conditions. This rendered the translation of uORF-containing mRNAs partially resistant to stress, as compared to the *control Fluc* mRNA without uORF, although the absolute values of Fluc activity were nevertheless lower in the arsenite-treated cells due to a dramatic reduction of the overall ribosome recruitment onto the mRNA 5′ end. However, in the case of *REI Rluc-Fluc* (where the contribution of leaky scanning and 43S sliding was excluded), we even observed an increase in the absolute values of Fluc activity, providing a basis for physiologically relevant effects. In this case, Fluc activity can also be considered as a proxy of an aberrant 3′ UTR translation, which may contribute to the stress response.

The phenomenon of translational resistance of some uORF-containing mRNA to eIF2-inactivating stresses has long been established (for a review, see [113,114]). The classic example of uORF-mediated translation control under stress conditions is the delayed reinitiation exemplified by yeast GCN4 and mammalian ATF4 mRNAs, which require at least two non-overlapping uORFs in the 5′ UTR [115,116,117]. However, the genome-wide systems approach revealed a number of stress-resistant mRNAs having only a single uORF (for review, see [102]). The suggested mechanism relies on an altered recognition of the uORF AUG codon and requires peculiar features of the involved uORF [118]. Although in the present study we used the reporter mRNA with a non-specific artificial uORF sequence, likewise for the bicistronic Rluc-Fluc mRNAs, we nevertheless observed the partial resistance of their translation to the stress-induced inhibition. Moreover, in a few previously reported cases, the uORF-dependent regulation clearly relies not exclusively on the AUG recognition, but on some other events occurring at the uORF stop codon (see [119,120] and references therein). Thus, we suggest that other molecular mechanisms may underlie the translational control of specific uORF-containing mRNAs under stress, including the altered spatial distribution of translation factors between subcellular compartments.

As in the case of translation termination, the observed changes in reinitiation rates may be attributed to either a decrease in cytosolic concentration or post-translational modifications of key components involved in the process. Previously, a similar up-regulation in *uORF-Fluc* and *REI-Fluc* translation was observed in yeast lacking eIF2D, MCT-1, or DENR orthologs [57]. Thus, we hypothesized that the recruitment of these factors to SGs that we observed in arsenite-treated cells led to their depletion from the cytosol and caused changes in the reporter activity. We used cells lacking G3BP1/2 that are unable to form the arsenite-induced microscopically visible SGs [86] to validate the hypothesis. We expected to find a partial reversion of the observed effects in these cells, as no recruiting SG compartment is formed in this case. However, similarly to the analysis of the stop-codon readthrough rate (see above), we instead observed an even more pronounced increase in reinitiation rate: ~2-fold as high as in the wt cells. Although our data do not support the “sequestration“ model, it is possible that the increased readthrough and reinitiation rates are not caused by the absence of SGs per se, but rather the absence of G3BP. As in the other studies, while ddG3BP1/2 cells are a useful model, they cannot distinguish between the direct effects of SG absence or G3BP absence. It could be that the absence of SGs and/or G3BP causes other changes, such as modifications of a translation machinery component(s), which could affect reinitiation rates. Finally, it is worth considering the possibility that increased readthrough and reinitiation rates could be advantageous under stress under conditions when SGs are not formed. Understanding the causes and consequences of the translation changes observed in ddG3BP1/2 cells is an important future research direction.

## 5. Conclusions

In summary, our study presents evidence that relocalization of translation termination and ribosome recycling/reinitiation factors to SGs and their partial depletion from the cytoplasm during stress coincides with elevated stop-codon readthrough and reinitiation rates. These spatial and functional changes may contribute significantly to reshaping the mammalian translatome under stress conditions.

## Data Availability

Not applicable.

## Supplementary Materials

The following supporting information can be downloaded at: https://www.mdpi.com/article/10.3390/cells12020259/s1, Figure S1: Rates of translation reinitiation and stop-codon readthrough are increased under conditions of arsenite-induced oxidative stress in HeLa cells; Figure S2: Translation termination factors are recruited to arsenite-induced SGs in EGFP-PABP expressing HeLa cells; Figure S3: Ribosome recycling factors are recruited to arsenite-induced SGs in EGFP-PABP expressing HeLa cells; Figure S4: Translation termination and ribosome recycling factors are partially recruited to SGs but not to PBs in wt U2OS cells; Figure S5: Localization of eRF1 in arsenite-treated ddG3BP1/2 cells stained for SG and PB marker proteins; Figure S6: eRF1 and eIF2D recruitment to SGs responds to puromycin and emetine treatment; Table S1: mRNA constructs used in this study [57,70,71].

## References

[1] Advani V.M., Ivanov P. (2019). Translational Control under Stress: Reshaping the Translatome. Bioessays.

[2] Leprivier G., Rotblat B., Khan D., Jan E., Sorensen P.H. (2015). Stress-mediated translational control in cancer cells. Biochim. Biophys. Acta.

[3] Janapala Y., Preiss T., Shirokikh N.E. (2019). Control of Translation at the Initiation Phase During Glucose Starvation in Yeast. Int. J. Mol. Sci..

[4] Buttgereit F., Brand M.D. (1995). A hierarchy of ATP-consuming processes in mammalian cells. Biochem. J..

[5] Young S.K., Wek R.C. (2016). Upstream Open Reading Frames Differentially Regulate Gene-specific Translation in the Integrated Stress Response. J. Biol. Chem..

[6] Andreev D.E., O’Connor P.B., Fahey C., Kenny E.M., Terenin I.M., Dmitriev S.E., Cormican P., Morris D.W., Shatsky I.N., Baranov P.V. (2015). Translation of 5’ leaders is pervasive in genes resistant to eIF2 repression. eLife.

[7] Lashkevich K.A., Dmitriev S.E. (2021). mRNA Targeting, Transport and Local Translation in Eukaryotic Cells: From the Classical View to a Diversity of New Concepts. Mol. Biol..

[8] Ivanov P., Kedersha N., Anderson P. (2019). Stress Granules and Processing Bodies in Translational Control. Cold Spring Harb. Perspect. Biol..

[9] Riggs C.L., Kedersha N., Ivanov P., Anderson P. (2020). Mammalian stress granules and P bodies at a glance. J. Cell Sci..

[10] Guzikowski A.R., Chen Y.S., Zid B.M. (2019). Stress-induced mRNP granules: Form and function of processing bodies and stress granules. Wiley Interdiscip. Rev. RNA.

[11] Back S.H. (2020). Roles of the Translation Initiation Factor eIF2alpha Phosphorylation in Cell Structure and Function. Cell Struct. Funct..

[12] Anderson P., Kedersha N. (2006). RNA granules. J. Cell Biol..

[13] Thomas M.G., Loschi M., Desbats M.A., Boccaccio G.L. (2011). RNA granules: The good, the bad and the ugly. Cell Signal..

[14] Protter D.S., Parker R. (2016). Principles and Properties of Stress Granules. Trends Cell Biol..

[15] Sorokin I.I., Vassilenko K.S., Terenin I.M., Kalinina N.O., Agol V.I., Dmitriev S.E. (2021). Non-Canonical Translation Initiation Mechanisms Employed by Eukaryotic Viral mRNAs. Biochemistry.

[16] Anderson P., Kedersha N. (2008). Stress granules: The Tao of RNA triage. Trends Biochem. Sci..

[17] Anderson P., Kedersha N., Ivanov P. (2015). Stress granules, P-bodies and cancer. Biochim. Biophys. Acta.

[18] Mazroui R., Huot M.E., Tremblay S., Filion C., Labelle Y., Khandjian E.W. (2002). Trapping of messenger RNA by Fragile X Mental Retardation protein into cytoplasmic granules induces translation repression. Hum. Mol. Genet..

[19] Kedersha N., Ivanov P., Anderson P. (2013). Stress granules and cell signaling: More than just a passing phase?. Trends Biochem. Sci..

[20] Fukuda T., Naiki T., Saito M., Irie K. (2009). hnRNP K interacts with RNA binding motif protein 42 and functions in the maintenance of cellular ATP level during stress conditions. Genes Cells.

[21] Kedersha N., Stoecklin G., Ayodele M., Yacono P., Lykke-Andersen J., Fritzler M.J., Scheuner D., Kaufman R.J., Golan D.E., Anderson P. (2005). Stress granules and processing bodies are dynamically linked sites of mRNP remodeling. J. Cell Biol..

[22] Cougot N., Babajko S., Seraphin B. (2004). Cytoplasmic foci are sites of mRNA decay in human cells. J. Cell Biol..

[23] Jain S., Wheeler J.R., Walters R.W., Agrawal A., Barsic A., Parker R. (2016). ATPase-Modulated Stress Granules Contain a Diverse Proteome and Substructure. Cell.

[24] Youn J.Y., Dunham W.H., Hong S.J., Knight J.D.R., Bashkurov M., Chen G.I., Bagci H., Rathod B., MacLeod G., Eng S.W.M. (2018). High-Density Proximity Mapping Reveals the Subcellular Organization of mRNA-Associated Granules and Bodies. Mol. Cell.

[25] Markmiller S., Soltanieh S., Server K.L., Mak R., Jin W., Fang M.Y., Luo E.C., Krach F., Yang D., Sen A. (2018). Context-Dependent and Disease-Specific Diversity in Protein Interactions within Stress Granules. Cell.

[26] Youn J.Y., Dyakov B.J.A., Zhang J., Knight J.D.R., Vernon R.M., Forman-Kay J.D., Gingras A.C. (2019). Properties of Stress Granule and P-Body Proteomes. Mol. Cell.

[27] Marmor-Kollet H., Siany A., Kedersha N., Knafo N., Rivkin N., Danino Y.M., Moens T.G., Olender T., Sheban D., Cohen N. (2020). Spatiotemporal Proteomic Analysis of Stress Granule Disassembly Using APEX Reveals Regulation by SUMOylation and Links to ALS Pathogenesis. Mol. Cell.

[28] Harvey R., Dezi V., Pizzinga M., Willis A.E. (2017). Post-transcriptional control of gene expression following stress: The role of RNA-binding proteins. Biochem. Soc. Trans..

[29] Kedersha N., Chen S., Gilks N., Li W., Miller I.J., Stahl J., Anderson P. (2002). Evidence that ternary complex (eIF2-GTP-tRNA(i)(Met))-deficient preinitiation complexes are core constituents of mammalian stress granules. Mol. Biol. Cell.

[30] Garcia-Moreno M., Sanz M.A., Pelletier J., Carrasco L. (2013). Requirements for eIF4A and eIF2 during translation of Sindbis virus subgenomic mRNA in vertebrate and invertebrate host cells. Cell. Microbiol..

[31] Li C.H., Ohn T., Ivanov P., Tisdale S., Anderson P. (2010). eIF5A promotes translation elongation, polysome disassembly and stress granule assembly. PLoS ONE.

[32] Fujimura K., Sasaki A.T., Anderson P. (2012). Selenite targets eIF4E-binding protein-1 to inhibit translation initiation and induce the assembly of non-canonical stress granules. Nucleic Acids Res..

[33] Grousl T., Ivanov P., Malcova I., Pompach P., Frydlova I., Slaba R., Senohrabkova L., Novakova L., Hasek J. (2013). Heat shock-induced accumulation of translation elongation and termination factors precedes assembly of stress granules in S. cerevisiae. PLoS ONE.

[34] Alkalaeva E.Z., Pisarev A.V., Frolova L.Y., Kisselev L.L., Pestova T.V. (2006). In vitro reconstitution of eukaryotic translation reveals cooperativity between release factors eRF1 and eRF3. Cell.

[35] Kisselev L.L., Frolova L. (1995). Termination of translation in eukaryotes. Biochem. Cell Biol..

[36] Hellen C.U.T. (2018). Translation Termination and Ribosome Recycling in Eukaryotes. Cold Spring Harb. Perspect. Biol..

[37] Dever T.E., Green R. (2012). The elongation, termination, and recycling phases of translation in eukaryotes. Cold Spring Harb. Perspect. Biol..

[38] Shoemaker C.J., Green R. (2011). Kinetic analysis reveals the ordered coupling of translation termination and ribosome recycling in yeast. Proc. Natl. Acad. Sci. USA.

[39] Khoshnevis S., Gross T., Rotte C., Baierlein C., Ficner R., Krebber H. (2010). The iron-sulphur protein RNase L inhibitor functions in translation termination. EMBO Rep..

[40] Janzen D.M., Geballe A.P. (2004). The effect of eukaryotic release factor depletion on translation termination in human cell lines. Nucleic Acids Res..

[41] Chauvin C., Salhi S., Le Goff C., Viranaicken W., Diop D., Jean-Jean O. (2005). Involvement of human release factors eRF3a and eRF3b in translation termination and regulation of the termination complex formation. Mol. Cell Biol..

[42] Reineke L.C., Dougherty J.D., Pierre P., Lloyd R.E. (2012). Large G3BP-induced granules trigger eIF2alpha phosphorylation. Mol. Biol Cell.

[43] Kimball S.R., Horetsky R.L., Ron D., Jefferson L.S., Harding H.P. (2003). Mammalian stress granules represent sites of accumulation of stalled translation initiation complexes. Am. J. Physiol. Cell Physiol..

[44] Kedersha N.L., Gupta M., Li W., Miller I., Anderson P. (1999). RNA-binding proteins TIA-1 and TIAR link the phosphorylation of eIF-2 alpha to the assembly of mammalian stress granules. J. Cell Biol..

[45] Kedersha N., Anderson P. (2009). Regulation of translation by stress granules and processing bodies. Prog. Mol. Biol. Transl. Sci..

[46] Hoyle N.P., Castelli L.M., Campbell S.G., Holmes L.E., Ashe M.P. (2007). Stress-dependent relocalization of translationally primed mRNPs to cytoplasmic granules that are kinetically and spatially distinct from P-bodies. J. Cell Biol..

[47] Buchan J.R., Yoon J.H., Parker R. (2011). Stress-specific composition, assembly and kinetics of stress granules in Saccharomyces cerevisiae. J. Cell Sci..

[48] Dang Y., Kedersha N., Low W.K., Romo D., Gorospe M., Kaufman R., Anderson P., Liu J.O. (2006). Eukaryotic initiation factor 2alpha-independent pathway of stress granule induction by the natural product pateamine A. J. Biol. Chem..

[49] Campbell S.G., Hoyle N.P., Ashe M.P. (2005). Dynamic cycling of eIF2 through a large eIF2B-containing cytoplasmic body: Implications for translation control. J. Cell Biol..

[50] Moutaoufik M.T., El Fatimy R., Nassour H., Gareau C., Lang J., Tanguay R.M., Mazroui R., Khandjian E.W. (2014). UVC-induced stress granules in mammalian cells. PLoS ONE.

[51] Low W.K., Dang Y., Schneider-Poetsch T., Shi Z., Choi N.S., Merrick W.C., Romo D., Liu J.O. (2005). Inhibition of eukaryotic translation initiation by the marine natural product pateamine A. Mol. Cell.

[52] Sanz M.A., Gonzalez Almela E., Carrasco L. (2017). Translation of Sindbis Subgenomic mRNA is Independent of eIF2, eIF2A and eIF2D. Sci. Rep..

[53] Pisarev A.V., Skabkin M.A., Pisareva V.P., Skabkina O.V., Rakotondrafara A.M., Hentze M.W., Hellen C.U., Pestova T.V. (2010). The role of ABCE1 in eukaryotic posttermination ribosomal recycling. Mol. Cell.

[54] Young D.J., Guydosh N.R., Zhang F., Hinnebusch A.G., Green R. (2015). Rli1/ABCE1 Recycles Terminating Ribosomes and Controls Translation Reinitiation in 3’UTRs In Vivo. Cell.

[55] Mancera-Martinez E., Brito Querido J., Valasek L.S., Simonetti A., Hashem Y. (2017). ABCE1: A special factor that orchestrates translation at the crossroad between recycling and initiation. RNA Biol..

[56] Skabkin M.A., Skabkina O.V., Dhote V., Komar A.A., Hellen C.U., Pestova T.V. (2010). Activities of Ligatin and MCT-1/DENR in eukaryotic translation initiation and ribosomal recycling. Genes Dev..

[57] Young D.J., Makeeva D.S., Zhang F., Anisimova A.S., Stolboushkina E.A., Ghobakhlou F., Shatsky I.N., Dmitriev S.E., Hinnebusch A.G., Guydosh N.R. (2018). Tma64/eIF2D, Tma20/MCT-1, and Tma22/DENR Recycle Post-termination 40S Subunits In Vivo. Mol. Cell.

[58] Schuller A.P., Green R. (2018). Roadblocks and resolutions in eukaryotic translation. Nat Rev Mol Cell Biol..

[59] Skabkin M.A., Skabkina O.V., Hellen C.U., Pestova T.V. (2013). Reinitiation and other unconventional posttermination events during eukaryotic translation. Mol. Cell.

[60] Dmitriev S.E., Terenin I.M., Andreev D.E., Ivanov P.A., Dunaevsky J.E., Merrick W.C., Shatsky I.N. (2010). GTP-independent tRNA delivery to the ribosomal P-site by a novel eukaryotic translation factor. J. Biol. Chem..

[61] Hinnebusch A.G. (2017). Structural Insights into the Mechanism of Scanning and Start Codon Recognition in Eukaryotic Translation Initiation. Trends Biochem. Sci..

[62] Reinert L.S., Shi B., Nandi S., Mazan-Mamczarz K., Vitolo M., Bachman K.E., He H., Gartenhaus R.B. (2006). MCT-1 protein interacts with the cap complex and modulates messenger RNA translational profiles. Cancer Res..

[63] Fleischer T.C., Weaver C.M., McAfee K.J., Jennings J.L., Link A.J. (2006). Systematic identification and functional screens of uncharacterized proteins associated with eukaryotic ribosomal complexes. Genes Dev..

[64] Lomakin I.B., Stolboushkina E.A., Vaidya A.T., Zhao C., Garber M.B., Dmitriev S.E., Steitz T.A. (2017). Crystal Structure of the Human Ribosome in Complex with DENR-MCT-1. Cell Rep..

[65] Weisser M., Schafer T., Leibundgut M., Bohringer D., Aylett C.H.S., Ban N. (2017). Structural and Functional Insights into Human Re-initiation Complexes. Mol. Cell.

[66] Costanzo M., Baryshnikova A., Bellay J., Kim Y., Spear E.D., Sevier C.S., Ding H., Koh J.L., Toufighi K., Mostafavi S. (2010). The genetic landscape of a cell. Science.

[67] Schleich S., Acevedo J.M., Clemm von Hohenberg K., Teleman A.A. (2017). Identification of transcripts with short stuORFs as targets for DENR*MCTS1-dependent translation in human cells. Sci. Rep..

[68] Schleich S., Strassburger K., Janiesch P.C., Koledachkina T., Miller K.K., Haneke K., Cheng Y.S., Kuchler K., Stoecklin G., Duncan K.E. (2014). DENR-MCT-1 promotes translation re-initiation downstream of uORFs to control tissue growth. Nature.

[69] Kedersha N., Anderson P. (2007). Mammalian stress granules and processing bodies. Methods Enzymol..

[70] Dmitriev S.E., Andreev D.E., Terenin I.M., Olovnikov I.A., Prassolov V.S., Merrick W.C., Shatsky I.N. (2007). Efficient translation initiation directed by the 900-nucleotide-long and GC-rich 5’ untranslated region of the human retrotransposon LINE-1 mRNA is strictly cap dependent rather than internal ribosome entry site mediated. Mol. Cell Biol..

[71] Ivanov A., Shuvalova E., Egorova T., Shuvalov A., Sokolova E., Bizyaev N., Shatsky I., Terenin I., Alkalaeva E. (2019). Polyadenylate-binding protein-interacting proteins PAIP1 and PAIP2 affect translation termination. J. Biol. Chem..

[72] Akulich K.A., Andreev D.E., Terenin I.M., Smirnova V.V., Anisimova A.S., Makeeva D.S., Arkhipova V.I., Stolboushkina E.A., Garber M.B., Prokofjeva M.M. (2016). Four translation initiation pathways employed by the leaderless mRNA in eukaryotes. Sci. Rep..

[73] Brostrom C.O., Prostko C.R., Kaufman R.J., Brostrom M.A. (1996). Inhibition of translational initiation by activators of the glucose-regulated stress protein and heat shock protein stress response systems. Role of the interferon-inducible double-stranded RNA-activated eukaryotic initiation factor 2alpha kinase. J. Biol. Chem..

[74] Bernstam L., Nriagu J. (2000). Molecular aspects of arsenic stress. J. Toxicol. Environ. Health B Crit. Rev..

[75] Ruiz-Ramos R., Lopez-Carrillo L., Rios-Perez A.D., De Vizcaya-Ruiz A., Cebrian M.E. (2009). Sodium arsenite induces ROS generation, DNA oxidative damage, HO-1 and c-Myc proteins, NF-kappaB activation and cell proliferation in human breast cancer MCF-7 cells. Mutat. Res..

[76] Brostrom C.O., Brostrom M.A. (1998). Regulation of translational initiation during cellular responses to stress. Prog. Nucleic Acid Res. Mol. Biol..

[77] Kozak M. (2002). Pushing the limits of the scanning mechanism for initiation of translation. Gene.

[78] Terenin I.M., Akulich K.A., Andreev D.E., Polyanskaya S.A., Shatsky I.N., Dmitriev S.E. (2016). Sliding of a 43S ribosomal complex from the recognized AUG codon triggered by a delay in eIF2-bound GTP hydrolysis. Nucleic Acids Res..

[79] Gunisova S., Hronova V., Mohammad M.P., Hinnebusch A.G., Valasek L.S. (2018). Please do not recycle! Translation reinitiation in microbes and higher eukaryotes. FEMS Microbiol. Rev..

[80] Balagopal V., Parker R. (2009). Polysomes, P bodies and stress granules: States and fates of eukaryotic mRNAs. Curr. Opin. Cell Biol..

[81] Aulas A., Fay M.M., Szaflarski W., Kedersha N., Anderson P., Ivanov P. (2017). Methods to Classify Cytoplasmic Foci as Mammalian Stress Granules. J. Vis. Exp..

[82] Buchan J.R., Muhlrad D., Parker R. (2008). P bodies promote stress granule assembly in Saccharomyces cerevisiae. J. Cell Biol..

[83] Dori D., Choder M. (2007). Conceptual modeling in systems biology fosters empirical findings: The mRNA lifecycle. PLoS ONE.

[84] Dmitriev S.E., Vladimirov D.O., Lashkevich K.A. (2020). A Quick Guide to Small-Molecule Inhibitors of Eukaryotic Protein Synthesis. Biochemistry.

[85] Kedersha N., Cho M.R., Li W., Yacono P.W., Chen S., Gilks N., Golan D.E., Anderson P. (2000). Dynamic shuttling of TIA-1 accompanies the recruitment of mRNA to mammalian stress granules. J. Cell Biol..

[86] Kedersha N., Panas M.D., Achorn C.A., Lyons S., Tisdale S., Hickman T., Thomas M., Lieberman J., McInerney G.M., Ivanov P. (2016). G3BP-Caprin1-USP10 complexes mediate stress granule condensation and associate with 40S subunits. J. Cell Biol..

[87] Matsuki H., Takahashi M., Higuchi M., Makokha G.N., Oie M., Fujii M. (2013). Both G3BP1 and G3BP2 contribute to stress granule formation. Genes Cells.

[88] Buchan J.R., Parker R. (2009). Eukaryotic stress granules: The ins and outs of translation. Mol. Cell.

[89] Hoshino S., Imai M., Kobayashi T., Uchida N., Katada T. (1999). The eukaryotic polypeptide chain releasing factor (eRF3/GSPT) carrying the translation termination signal to the 3’-Poly(A) tail of mRNA. Direct association of erf3/GSPT with polyadenylate-binding protein. J. Biol. Chem..

[90] Uchida N., Hoshino S., Imataka H., Sonenberg N., Katada T. (2002). A novel role of the mammalian GSPT/eRF3 associating with poly(A)-binding protein in Cap/Poly(A)-dependent translation. J. Biol. Chem..

[91] Roque S., Cerciat M., Gaugue I., Mora L., Floch A.G., de Zamaroczy M., Heurgue-Hamard V., Kervestin S. (2015). Interaction between the poly(A)-binding protein Pab1 and the eukaryotic release factor eRF3 regulates translation termination but not mRNA decay in Saccharomyces cerevisiae. RNA.

[92] Wan C., Borgeson B., Phanse S., Tu F., Drew K., Clark G., Xiong X., Kagan O., Kwan J., Bezginov A. (2015). Panorama of ancient metazoan macromolecular complexes. Nature.

[93] Li X., Rayman J.B., Kandel E.R., Derkatch I.L. (2014). Functional role of Tia1/Pub1 and Sup35 prion domains: Directing protein synthesis machinery to the tubulin cytoskeleton. Mol. Cell.

[94] Urakov V.N., Mitkevich O.V., Dergalev A.A., Ter-Avanesyan M.D. (2018). The Pub1 and Upf1 Proteins Act in Concert to Protect Yeast from Toxicity of the [PSI(+)] Prion. Int. J. Mol. Sci..

[95] Mateju D., Eichenberger B., Voigt F., Eglinger J., Roth G., Chao J.A. (2020). Single-Molecule Imaging Reveals Translation of mRNAs Localized to Stress Granules. Cell.

[96] Moon S.L., Morisaki T., Khong A., Lyon K., Parker R., Stasevich T.J. (2019). Multicolour single-molecule tracking of mRNA interactions with RNP granules. Nat. Cell Biol..

[97] Lashkevich K.A., Shlyk V.I., Kushchenko A.S., Gladyshev V.N., Alkalaeva E.Z., Dmitriev S.E. (2020). CTELS: A Cell-Free System for the Analysis of Translation Termination Rate. Biomolecules.

[98] Lawson M.R., Lessen L.N., Wang J., Prabhakar A., Corsepius N.C., Green R., Puglisi J.D. (2021). Mechanisms that ensure speed and fidelity in eukaryotic translation termination. Science.

[99] Katz M.J., Gandara L., De Lella Ezcurra A.L., Wappner P. (2016). Hydroxylation and translational adaptation to stress: Some answers lie beyond the STOP codon. Cell Mol. Life Sci..

[100] Rodnina M.V., Korniy N., Klimova M., Karki P., Peng B.Z., Senyushkina T., Belardinelli R., Maracci C., Wohlgemuth I., Samatova E. (2020). Translational recoding: Canonical translation mechanisms reinterpreted. Nucleic Acids Res..

[101] Bersch K., Lobos Matthei I., Thoms S. (2018). Multiple Localization by Functional Translational Readthrough. Subcell. Biochem..

[102] Andreev D.E., O’Connor P.B., Loughran G., Dmitriev S.E., Baranov P.V., Shatsky I.N. (2017). Insights into the mechanisms of eukaryotic translation gained with ribosome profiling. Nucleic Acids Res..

[103] Gerashchenko M.V., Lobanov A.V., Gladyshev V.N. (2012). Genome-wide ribosome profiling reveals complex translational regulation in response to oxidative stress. Proc. Natl. Acad. Sci. USA.

[104] Andreev D.E., O’Connor P.B., Zhdanov A.V., Dmitriev R.I., Shatsky I.N., Papkovsky D.B., Baranov P.V. (2015). Oxygen and glucose deprivation induces widespread alterations in mRNA translation within 20 minutes. Genome Biol..

[105] Wang D., Zavadil J., Martin L., Parisi F., Friedman E., Levy D., Harding H., Ron D., Gardner L.B. (2011). Inhibition of nonsense-mediated RNA decay by the tumor microenvironment promotes tumorigenesis. Mol. Cell Biol..

[106] Kano S., Nishida K., Kurebe H., Nishiyama C., Kita K., Akaike Y., Kajita K., Kurokawa K., Masuda K., Kuwano Y. (2014). Oxidative stress-inducible truncated serine/arginine-rich splicing factor 3 regulates interleukin-8 production in human colon cancer cells. Am. J. Physiol. Cell Physiol..

[107] Chen Z.Q., Dong J., Ishimura A., Daar I., Hinnebusch A.G., Dean M. (2006). The essential vertebrate ABCE1 protein interacts with eukaryotic initiation factors. J. Biol. Chem..

[108] Heuer A., Gerovac M., Schmidt C., Trowitzsch S., Preis A., Kotter P., Berninghausen O., Becker T., Beckmann R., Tampe R. (2017). Structure of the 40S-ABCE1 post-splitting complex in ribosome recycling and translation initiation. Nat. Struct. Mol. Biol..

[109] Dong J., Lai R., Nielsen K., Fekete C.A., Qiu H., Hinnebusch A.G. (2004). The essential ATP-binding cassette protein RLI1 functions in translation by promoting preinitiation complex assembly. J. Biol. Chem..

[110] Makeeva D.S., Lando A.S., Anisimova A., Egorov A.A., Logacheva M.D., Penin A.A., Andreev D.E., Sinitcyn P.G., Terenin I.M., Shatsky I.N. (2019). Translatome and transcriptome analysis of TMA20 (MCT-1) and TMA64 (eIF2D) knockout yeast strains. Data Brief..

[111] Janich P., Arpat A.B., Castelo-Szekely V., Lopes M., Gatfield D. (2015). Ribosome profiling reveals the rhythmic liver translatome and circadian clock regulation by upstream open reading frames. Genome Res..

[112] Haas M.A., Ngo L., Li S.S., Schleich S., Qu Z., Vanyai H.K., Cullen H.D., Cardona-Alberich A., Gladwyn-Ng I.E., Pagnamenta A.T. (2016). De Novo Mutations in DENR Disrupt Neuronal Development and Link Congenital Neurological Disorders to Faulty mRNA Translation Re-initiation. Cell Rep..

[113] Zhang H., Wang Y., Lu J. (2019). Function and Evolution of Upstream ORFs in Eukaryotes. Trends Biochem. Sci..

[114] Chen H.H., Tarn W.Y. (2019). uORF-mediated translational control: Recently elucidated mechanisms and implications in cancer. RNA Biol..

[115] Hinnebusch A.G. (1993). Gene-specific translational control of the yeast GCN4 gene by phosphorylation of eukaryotic initiation factor 2. Mol. Microbiol..

[116] Vattem K.M., Wek R.C. (2004). Reinitiation involving upstream ORFs regulates ATF4 mRNA translation in mammalian cells. Proc Natl. Acad. Sci. USA.

[117] Lu P.D., Harding H.P., Ron D. (2004). Translation reinitiation at alternative open reading frames regulates gene expression in an integrated stress response. J. Cell Biol..

[118] Andreev D.E., Arnold M., Kiniry S.J., Loughran G., Michel A.M., Rachinskii D., Baranov P.V. (2018). TASEP modelling provides a parsimonious explanation for the ability of a single uORF to derepress translation during the integrated stress response. eLife.

[119] Akulich K.A., Sinitcyn P.G., Makeeva D.S., Andreev D.E., Terenin I.M., Anisimova A.S., Shatsky I.N., Dmitriev S.E. (2019). A novel uORF-based regulatory mechanism controls translation of the human MDM2 and eIF2D mRNAs during stress. Biochimie.

[120] Ait Ghezala H., Jolles B., Salhi S., Castrillo K., Carpentier W., Cagnard N., Bruhat A., Fafournoux P., Jean-Jean O. (2012). Translation termination efficiency modulates ATF4 response by regulating ATF4 mRNA translation at 5’ short ORFs. Nucleic Acids Res..

