# Supplementary material for: Relocalization of Translation Termination and Ribosome Recycling Factors to Stress Granules Coincides with Elevated Stop-Codon Readthrough and Reinitiation Rates upon Oxidative Stress
Source: Cells. 2023 Jan 8;12(2):259. doi: 10.3390/cells12020259 (PMC9856671; doi:10.3390/cells12020259)
Supplement: Supplementary file 1 [file cells-12-00259-s001.zip › Makeeva_Supplementary_materials.pdf]

cells **MDPI**

Relocalization of translation termination and ribosome recycling factors to stress granules coincides with elevated stop-codon readthrough and reinitiation rates upon oxidative stress

Desislava S. Makeeva, Claire L. Riggs, Anton V. Burakov, Pavel A. Ivanov, Artem S. Kushchenko, Dmitri A. Bykov, Vladimir I. Popenko, Vladimir S. Prassolov, Pavel V. Ivanov, and Sergey E. Dmitriev

Supplementary materials

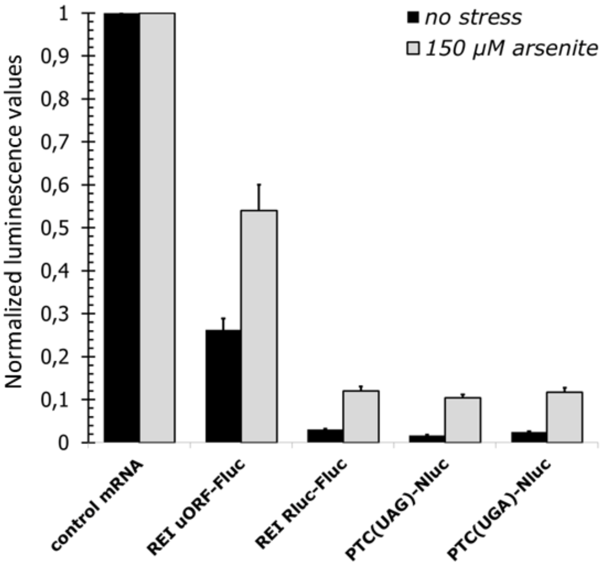

Figure S1. Rates of translation reinitiation and stop-codon readthrough are increased under conditions of arsenite-induced oxidative stress in HeLa cells. Reporter mRNAs (shown in Figure 1) were transfected in arsenite-treated or non-treated HeLa cells. 2 h after transfection, cells were lysed, luciferase activities were measured and normalized as described in the legend to Figure 1. Translation efficiencies of the reporter mRNAs, divided to those of the corresponding control transcripts, in untreated or 150 μM arsenite-treated cells. The mean values (±SD) of at least three independent experiments are shown. The absolute values were roughly similar to those indicated in the legend to Figure 1 (and were at least two orders of magnitude above the background).

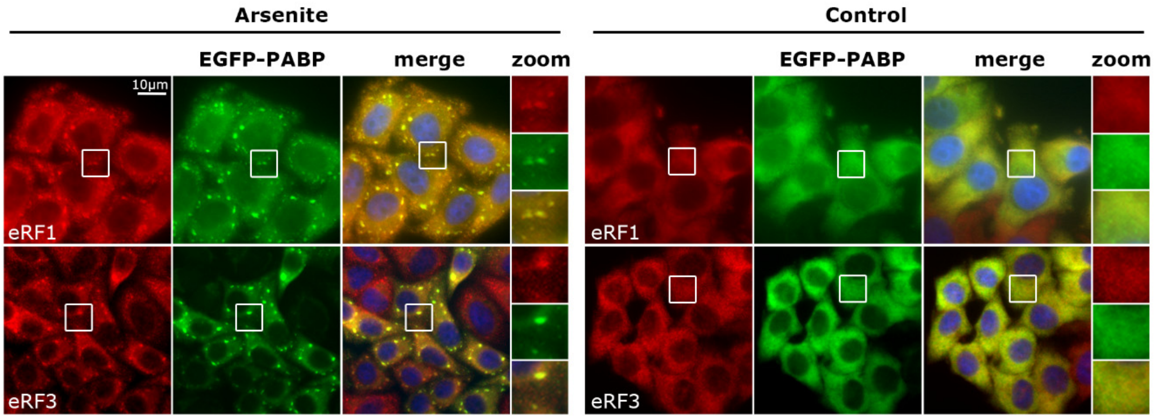

Figure S2. Translation termination factors are recruited to arsenite-induced SGs in EGFP-PABP expressing HeLa cells. HeLa cells expressing a EGFP-PABPC1 fusion (green channel) were treated with 250 μM sodium arsenite for 1 h (left panel) or left untreated (right), followed by fixation and immunostaining with anti-eRF1 or anti-eRF3 antibodies (red channel), as indicated. A few representative cells are shown; boxes indicate zoomed areas. All large images are of the same magnification (scale bar: 10 μm).

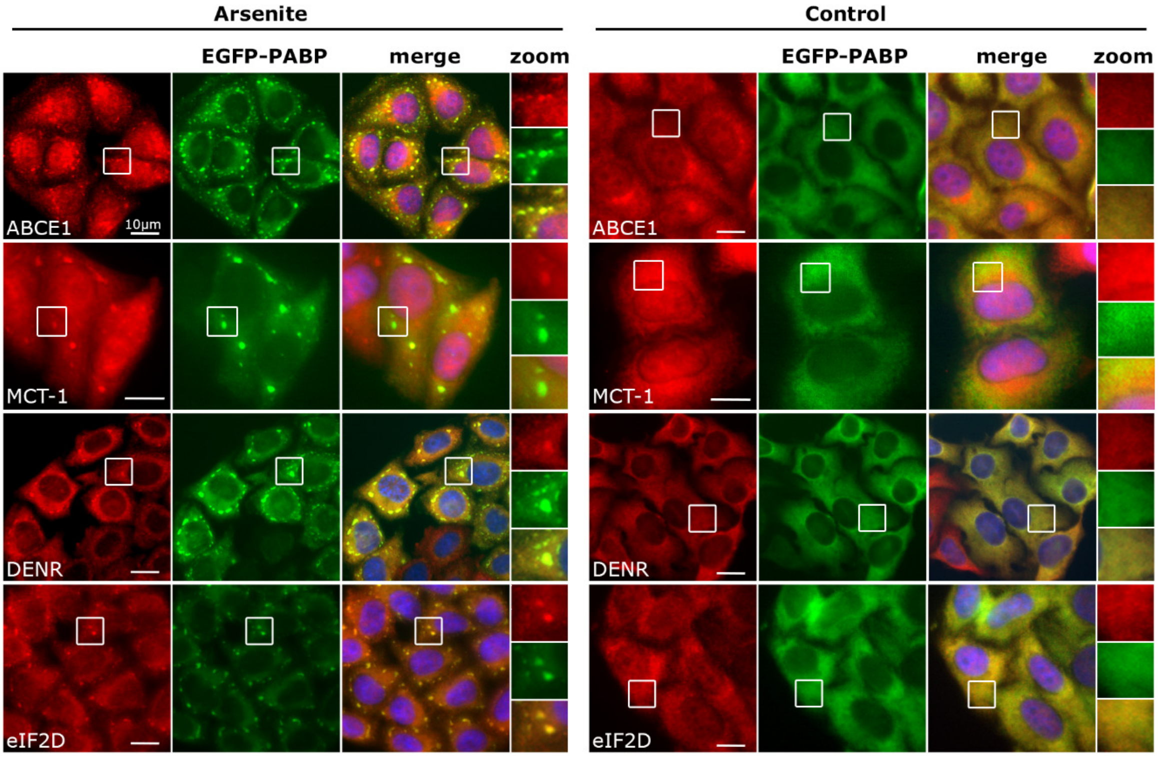

Figure S3. Ribosome recycling factors are recruited to arsenite-induced SGs in EGFP-PABP expressing HeLa cells. HeLa cells expressing a EGFP-PABPC1 fusion (green channel) were treated with 250 μM sodium arsenite for 1 h (left panel) or left untreated (right), followed by fixation and immunostaining with antibodies to indicated proteins (red channel). A few representative cells are shown; boxes indicate zoomed areas. All large images are of the same magnification (scale bar: 10 μm; note that the panels with MCT-1 have a higher magnification, see the bars).

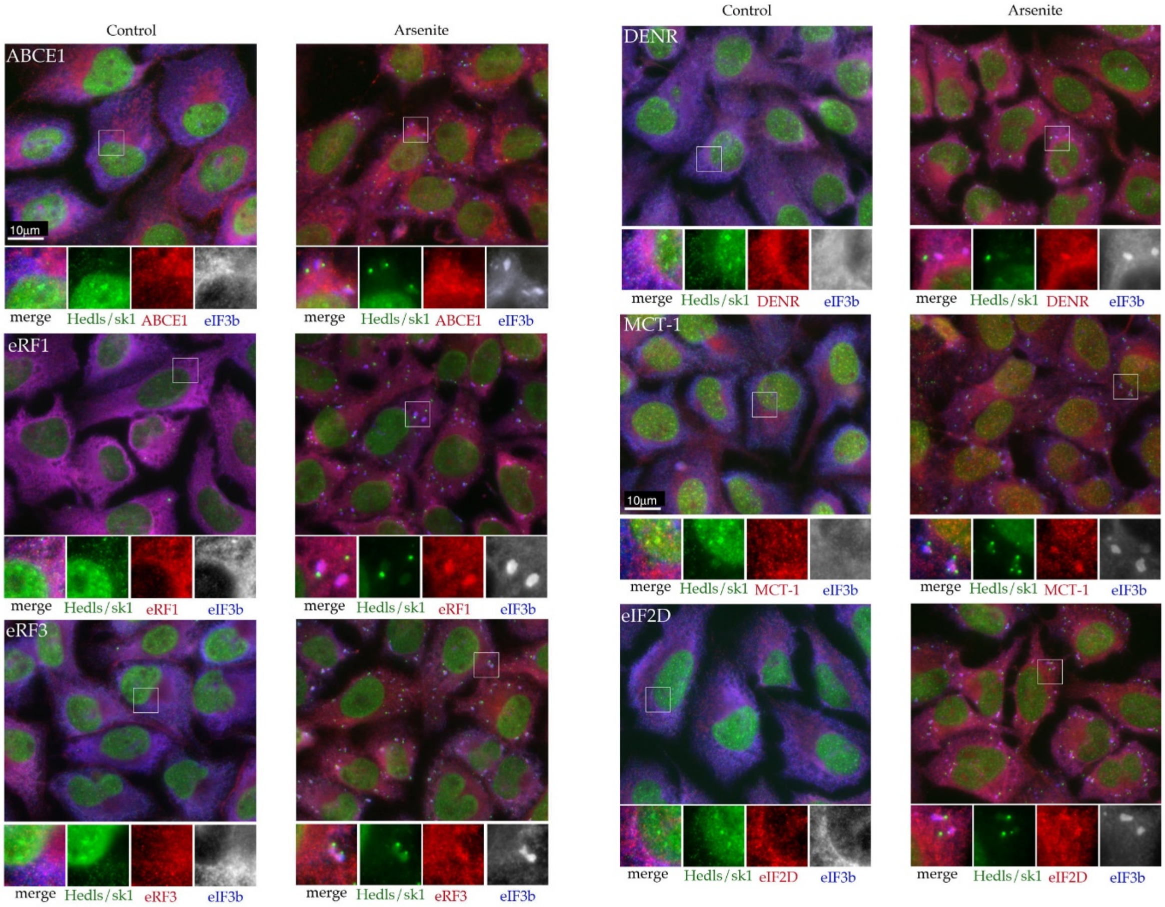

Figure S4. Translation termination and ribosome recycling factors are partially recruited to SGs but not to PBs in wt U2OS cells. U2OS cells were treated with 250 µM sodium arsenite for 1 h or left untreated, as indicated, followed by fixation and immunostaining with antibodies to indicated proteins of interest along with an SG marker (eIF3b) and a PB marker (Hedls/sk1). White boxes indicate representative areas of cells, which are shown magnified below. All large images are of the same magnification (scale bar: 10 µm).

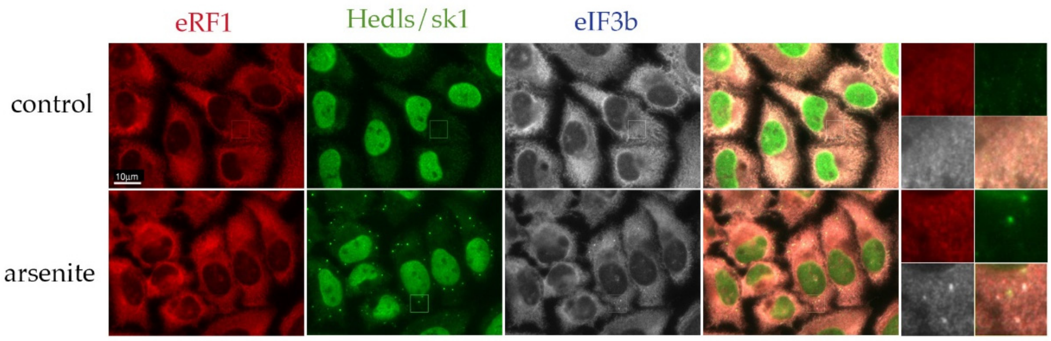

Figure S5. Localization of eRF1 in arsenite-treated ddG3BP1/2 cells stained for SG and PB marker proteins. U2OS ddG3BP1/2 cells were treated with 250 µM sodium arsenite for 1 h and immunostained for eRF1 along with an SG marker (eIF3b) and a PB marker (Hedls/sk1). Boxes indicate zoomed areas.

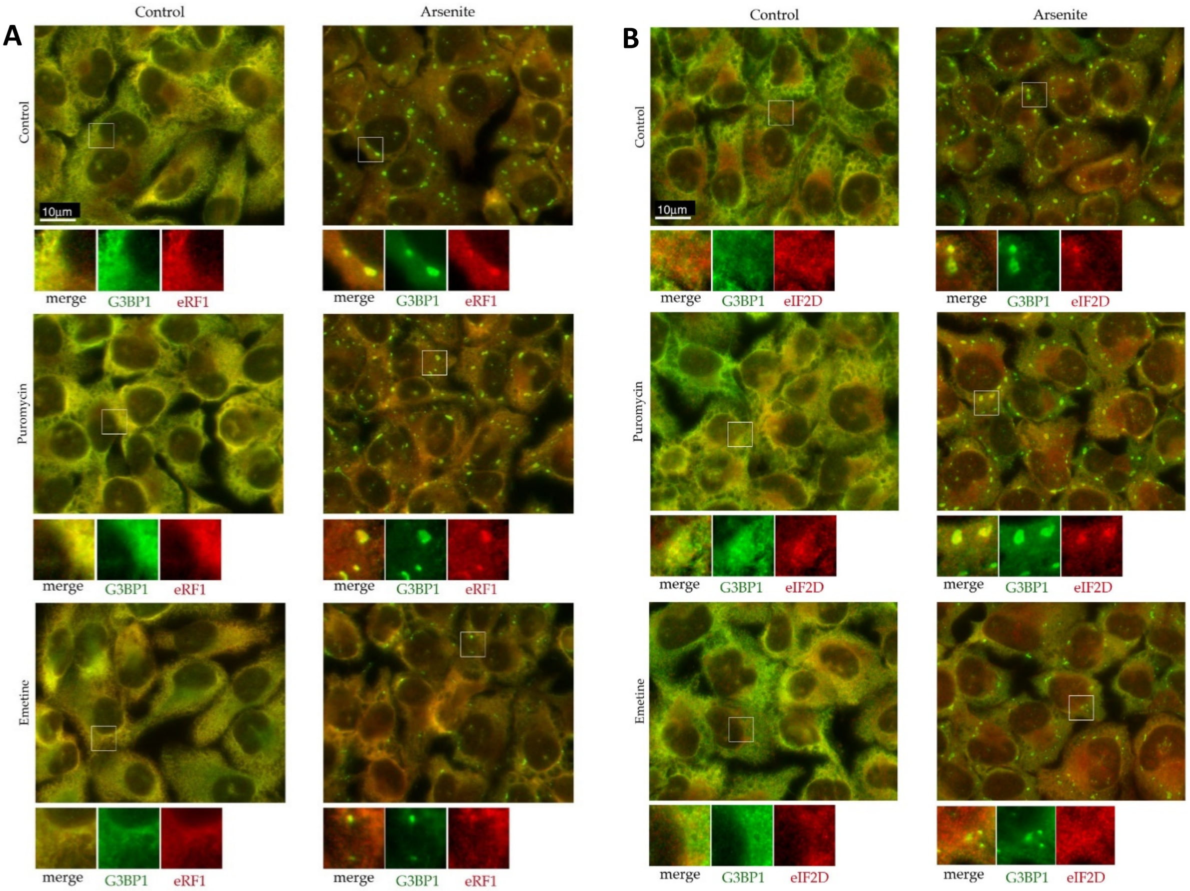

Figure S6. eRF1 and eIF2D recruitment to SGs responds to puromycin and emetine treatment. U2OS cells were treated with 250 μM sodium arsenite for 90 min or left untreated. 30 min later, cells were treated with either puromycin (10 μg/ml) to promote SG assembly, or emetine (10 μg/ml) to partially disassemble SGs. Treated cells were immunostained for G3BP1 (an SG marker) and either eRF1 or eIF2D. White boxes indicate representative areas of cells, which are shown magnified below. All large images are of the same magnification (scale bar: 10 μm).

Table S1. mRNA constructs used in this study. Nucleotide sequences of the most important mRNA regions are shown. The names of the constructs used in the original studies (see references) are given in the second column.

Construct	Source	Partial mRNA sequence
Fluc	[1] (no_uORFluc)	5′ UTR and Fluc start codon and CDS:<br>GGAACAACAACAACAACAAAAGUACACAAACACACUGAUGGAAGAC-<Fluc>
REI uORF-Fluc	[1] (uORF1luc)	5′ UTR, **uORF**, stop-start, and Fluc CDS:<br>GGAACAACAACAACAACAAA**AUGACACAAACACACUGA**UGGAAGAC-<Fluc>
REI Rluc-Fluc	[1] (RFluc2)	Rluc-Fluc junction, stop-start is boxed:<br><Rluc>-GAACAAGAGGAAUUAUGAAAGAAGAC-<Fluc>
Control Rluc-Fluc	[1] (RF1luc1fus)	Rluc-Fluc junction:<br><Rluc>-GAACAAUGGAUCCUAAUAACACACAACACCGGAGCAACUAUGAAAGAAGAC-<Fluc>
Rluc	[2] (pSV40-Rluc)	5′ UTR and Rluc start codon:<br>GGCUAGGCUUGGCAUUCCGGUACUGAAUUGAUAUCAAGCUCUAGCCACCAUG-<Rluc>
PTC(UAG) -Nluc	[3] (pNL-globin_ UAG_AUA)	β-globin-Nluc junction, PTC is boxed:<br><β-Glo>-GCCGUUACUUAGGAUAAUACCAUAGUCUUCACACUC-<Nluc>
PTC(UGA) -Nluc	[3] (pNL-globin_ UGA_AUA)	β-globin-Nluc junction, PTC is boxed:<br><β-Glo>-GCCGUUACUUGACUAGUAACCAUAGUCUUCACACUC-<Nluc>
Nluc	[3] (pNL-globin_ AAA_AUA)	β-globin-Nluc junction:<br><β-Glo>-GCCGUUACUAAAGGGCUGACCAUAGUCUUCACACUC-<Nluc>

References

1. Young, D.J.; Makeeva, D.S.; Zhang, F.; Anisimova, A.S.; Stolboushkina, E.A.; Ghobakhlou, F.; Shatsky, I.N.; Dmitriev, S.E.; Hinnebusch, A.G.; Guydosh, N.R. Tma64/eIF2D, Tma20/MCT-1, and Tma22/DENR Recycle Post-termination 40S Subunits In Vivo. *Mol Cell*. **2018**, *71*, 761-774 e5.
2. Dmitriev, S.E.; Andreev, D.E.; Terenin, I.M.; Olovnikov, I.A.; Prassolov, V.S.; Merrick, W.C.; Shatsky, I.N. Efficient translation initiation directed by the 900-nucleotide-long and GC-rich 5' untranslated region of the human retrotransposon LINE-1 mRNA is strictly cap dependent rather than internal ribosome entry site mediated. *Mol Cell Biol*. **2007**, *27*, 4685-97.
3. Ivanov, A.; Shuvalova, E.; Egorova, T.; Shuvalov, A.; Sokolova, E.; Bizyaev, N.; Shatsky, I.; Terenin, I.; Alkalaeva, E. Polyadenylate-binding protein-interacting proteins PAIP1 and PAIP2 affect translation termination. *J Biol Chem*. **2019,** *294*, 8630-8639.
